# Targeting Liver Sinusoidal Endothelial Cells: An Attractive Therapeutic Strategy to Control Inflammation in Nonalcoholic Fatty Liver Disease

**DOI:** 10.3389/fphar.2021.655557

**Published:** 2021-04-15

**Authors:** Xue-Kai Wang, Zong-Gen Peng

**Affiliations:** ^1^ CAMS Key Laboratory of Antiviral Drug Research, Institute of Medicinal Biotechnology, Chinese Academy of Medical Sciences and Peking Union Medical College, Beijing, China; ^2^ Key Laboratory of Biotechnology of Antibiotics, National Health and Family Planning Commission, Institute of Medicinal Biotechnology, Chinese Academy of Medical Sciences and Peking Union Medical College, Beijing, China; ^3^ Beijing Key Laboratory of Antimicrobial Agents, Institute of Medicinal Biotechnology, Chinese Academy of Medical Sciences and Peking Union Medical College, Beijing, China

**Keywords:** nonalcoholic fatty liver disease, liver sinusoidal endothelial cell, inflammation, capillarization, endothelial dysfunction, therapeutic strategy

## Abstract

Nonalcoholic fatty liver disease (NAFLD), especially its advanced stage nonalcoholic steatohepatitis (NASH), has become a threatened public health problem worldwide. However, no specific drug has been approved for clinical use to treat patients with NASH, though there are many promising candidates against NAFLD in the drug development pipeline. Recently, accumulated evidence showed that liver sinusoidal endothelial cells (LSECs) play an essential role in the occurrence and development of liver inflammation in patients with NAFLD. LSECs, as highly specialized endothelial cells with unique structure and anatomical location, contribute to the maintenance of liver homeostasis and could be a promising therapeutic target to control liver inflammation of NAFLD. In this review, we outline the pathophysiological roles of LSECs related to inflammation of NAFLD, highlight the pro-inflammatory and anti-inflammatory effects of LSECs, and discuss the potential drug development strategies against NAFLD based on targeting to LSECs.

## Introduction

Nonalcoholic fatty liver disease (NAFLD), now proposed changing to metabolic-associated fatty liver disease (MAFLD), has gradually become one of the most common liver diseases in the world (Younossi et al., 2016; Eslam et al., 2020). Technically, NAFLD is divided into nonalcoholic fatty liver (NAFL) and nonalcoholic steatohepatitis (NASH) according to pathological conditions (Stefan et al., 2019). The main feature of NAFL is at least 5% of liver steatosis without hepatocyte damage in the form of hepatocyte ballooning, whereas NASH mainly manifests more than 5% of liver steatosis with inflammation and hepatocyte damage (Stefan et al., 2019). Typically, NASH is often accompanied with liver fibrosis and may highly develop into cirrhosis and even hepatocellular carcinoma (Younossi, 2019). In recent years, NASH has gradually become a leading cause of liver transplantation among adults in European and American countries (Wong et al., 2015; Estes et al., 2018; Younossi, 2019). However, despite the high incidence and severity of NASH, no specific drug is currently approved and existing treatment methods are only aimed at symptomatic treatment rather than mechanism-based (Friedman et al., 2018).

Inflammation is one of the major engines of NASH progression (Schuster et al., 2018). Although a moderate and resolved inflammatory response contributes to homeostasis remodeling and tissue repair and thereby has hepatoprotective effects, inflammation in NASH is persistent, which leads to hepatocyte death and liver parenchymal damage (Brenner et al., 2013). Furthermore, uninterrupted low-level inflammation can cause hepatic stellate cells (HSCs) to activate and differentiate into myofibroblasts. The activated myofibroblasts release a large amount of extracellular matrix, which are rich in collagen fibers, to extracellular space and eventually result in hepatic fibrosis or cirrhosis (Schuster et al., 2018; Schwabe et al., 2020). Therefore, understanding the mechanisms of occurrence and development of inflammation in NASH is of the utmost importance for better controlling inflammation, which is essential to prevent, alleviate, and even reverse fibrosis.

Vascular endothelium is located at the junction of circulating blood and peripheral tissues. In addition to acting as a physical barrier, vascular endothelium participates in various pathophysiological processes, including inflammation, angiogenesis, vascular tone regulation, platelet function regulation, and metabolic homeostasis (Chiu and Chien, 2011; Pi et al., 2018). Hepatic sinusoidal endothelium, as a physical barrier controlling material exchanges between liver parenchyma and circulation, is constituted of liver sinusoidal endothelial cells (LSECs) which are highly specialized endothelial cells and the most abundant liver non-parenchymal cells (Brunt et al., 2014). Due to their unique anatomical location, LSECs play a critical role in the pathophysiological activities of the liver. Recently accumulated evidence showed that LSECs play an essential role in the occurrence and progression of liver inflammation in NAFLD. In this review, we briefly summarize the pathophysiological role of LSECs related to liver inflammation, highlight the occurrence and development mechanisms of inflammation of NAFLD associated with LSECs, and finally discuss the potential drug development strategies against NAFLD based on targeting to LSECs.

## The Structure and Biological Function of Liver Sinusoidal Endothelial Cells

Due to their unique structure and anatomical location, LSECs play important physiological roles in the maintenance of liver homeostasis, including substance exchange, blood flow regulation, high endocytic capacity, and immune regulation.

### Unique Structure and Biological Function of Liver Sinusoidal Endothelial Cells in Substance Exchange

**FIGURE 1:**
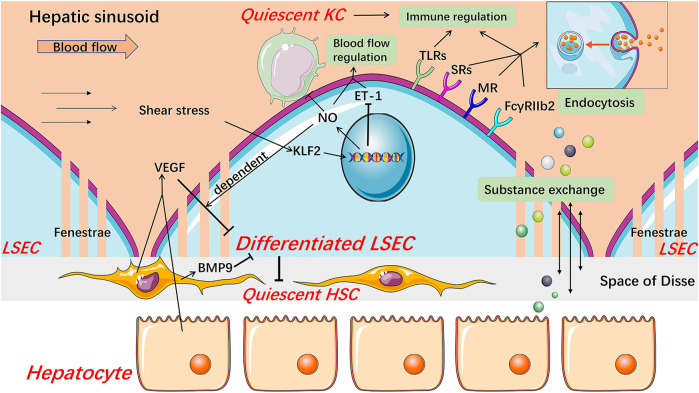
Structure and major physiological function of LSECs. Healthy differentiated LSECs, characterized by the fenestrated phenotype and lack of basement membrane, are located at the junction of circulating blood and liver parenchyma, which state is maintained by VEGF from hepatocytes and HSCs in an LSEC-derived NO-dependent manner and BMP9 from HSCs. LSECs increase NO production and reduce ET-1 expression through KLF2 in response to the shear stress in hepatic sinusoids, thereby regulating blood flow. NO also keeps KCs and HSCs quiescent. LSECs fenestrae constitute a sieve plate that makes LSECs highly permeable to better exchange substance between liver parenchyma and blood. The expression of SRs, MR, and FcγRIIb2 endows LSECs with high endocytic capacity. These receptors, along with TLRs, are related to the immunoregulatory function of LSECs which contributes to the immune clearance and antigen presentation of LSECs. *BMP9* Bone morphogenetic protein 9, *ET-1* endothelin-1, *FcγRIIb2* Fc gamma-receptor IIb2, *HSC* hepatic stellate cell, *KC* Kupffer cell, *KLF2* Kruppel-like factor 2, *LSEC* liver sinusoidal endothelial cell, *MR* mannose receptor, *NO* nitric oxide, *SRs* scavenger receptors, *TLRs* Toll-like receptors, *VEGF* vascular endothelial growth factor.

LSECs are unique in structure and function distinguished from other liver vascular endothelial cells. From an evolutionary point, the hepatic sinusoids are derived from the capillary vessels of the septum transversum and have a fenestrated phenotype acquired during 10 and 20 gestation weeks, which are different from the portal vessels that are developed from the vitelline veins (Gouysse et al., 2002; Geraud et al., 2017). In terms of structure and function, the specificity of LSECs is an adaptation to local microenvironment determined by anatomical location (Potente and Makinen, 2017). LSECs are located at an interface between the liver parenchyma and the mixed blood from the hepatic artery (30%) and portal vein (70%) (Poisson et al., 2017). On the sinusoidal side, LSECs are exposed to substances circulated in the blood, such as abundant nutrients, hormones, bile acids, and oxygen. While on the abluminal side, LSECs directly communicate with HSCs and hepatocytes that are critical for glycolipid metabolism. Therefore, the distinguished location feature assigns LSECs with the possibility of excellent substance exchange capacity (Poisson et al., 2017). Under normal conditions, LSECs have hallmarks characterized as the fenestrated phenotype, lack of basement membrane, and absence of diaphragm (Figure 1), and the unique structure made LSECs the most permeable endothelial cells in mammals, which is of significance for achieving more substance exchanges between liver parenchyma and blood to better match the metabolic function of the liver (Poisson et al., 2017). The fenestrae of LSECs, with a diameter of 50 ∼150 nm, connects the space of Disse with the sinusoidal side, allowing lipoproteins, chylomicron remnants (a small lipoprotein with a diameter of about 30∼80 nm that is decomposed by chylomicrons by lipoprotein lipase on the endothelial membrane of peripheral capillaries), and other macromolecules to enter the space of Disse from the circulating blood and then to be utilized by hepatocytes (Carpenter et al., 2005; Hilmer et al., 2005; Cogger et al., 2006). Besides, LSEC’s fenestrae are organized in clusters termed as sieve plates, in which the size and number of fenestrae vary in different positions and can be changed dynamically depending on physiological states such as fasting or pathological states (Wisse et al., 1985; O’Reilly et al., 2010; Xie et al., 2010; DeLeve, 2015; Monkemoller et al., 2015; Zapotoczny et al., 2019). Overall, due to their location features, structural changes, and high permeability, LSECs establish a differential and selective barrier that can be adjusted according to the changes of pathophysiological environment.

### Regulation of Liver Sinusoidal Endothelial Cells in Blood Flow

As endothelial cells, LSECs also play a role in regulating blood flow. Nitric oxide (NO) and endothelin-1 (ET-1), mainly produced by LSECs under normal conditions, are the two most powerful vasoactive substances in the liver, which causes dilation and contraction of blood vessels, respectively (Rieder et al., 1991; Shah et al., 1997). Under physiological conditions, in responding to the shear stress that is a friction between the vascular endothelium and blood flow, LSECs increase NO production and down-regulate ET-1 expression, which are mediated by Kruppel-like factor 2 (KLF2), an endothelial-specific transcription factor and a crucial anti-angiogenic factor (Davies, 1995; Shah et al., 1997; Parmar et al., 2006; Zeng et al., 2015). Meanwhile, healthy LSECs maintain HSCs and Kupffer cells (KCs) quiescence through NO-dependent pathways (Deleve et al., 2008; Tateya et al., 2011). These results indicate that LSECs may participate in inflammation and fibrosis under pathological conditions via NO signaling. Besides, carbon monoxide and the metabolites of the cyclooxygenase pathway, such as prostacyclin, are also associated with the regulation of blood flow in the hepatic sinusoids (Fernandez, 2015). Therefore, this regulation process involved with LSECs not only copes with the circadian change of hepatic blood flow mainly caused by digestion but also participates in maintaining the hepatic homeostasis, including inhibiting the occurrence of inflammation and fibrosis under pathophysiological condition.

### High Endocytic Capacity of Liver Sinusoidal Endothelial Cells

LSECs possess the strongest potential ability of endocytosis in the human body, which is also an outstanding feature of LSECs (Smedsrod et al., 2009). Based on the dual-cell principle of waste clearance, the cells responsible for cleaning up circulating waste are divided into two types. One is professional phagocytes represented by macrophages which mainly clean up larger particles (>0.5 μm) by phagocytosis. The other is professional pinocytes represented by the scavenger endothelial cells, including LSECs, which are responsible for cleaning soluble macromolecules and smaller particles by endocytosis (Sorensen et al., 2012). Accordingly, LSECs express a variety of endocytosis receptors, including scavenger receptor (SR), mannose receptor (MR), and Fc gamma-receptor IIb2 (FcγRIIb2). SRs, including SR-A, SR-B, and SR-H (stabilin-1 and stabilin-2), are expressed on normal LSECs, and stabilin-1 and stabilin-2 are thought to play a major role (Hughes et al., 1995; McCourt et al., 1999; Zhou et al., 2000; Adachi and Tsujimoto, 2002; Malerod et al., 2002; Politz et al., 2002; Hansen et al., 2005; Sorensen et al., 2012). Stabilin-1 and stabilin-2 mediate uptake and degradation of modified proteins and lipoproteins such as advanced glycation-end products-albumin and oxidized low-density lipoproteins (ox-LDL), extracellular matrix macromolecules, protein turnover by-products including hyaluronan, heparin, and chondroitin sulfate (Sorensen et al., 2012). MR mediates the uptake of a wide range of endogenous glycoproteins and microbial glycans, the recruitment of lysosomal enzymes, and the endocytosis of other tissue turnover waste products including collagen alpha chains, tissue plasminogen activator (Stahl and Ezekowitz, 1998; Lee et al., 2002; McGreal et al., 2004; Sorensen et al., 2012). In particular, the expression and activity of MR in the liver are regulated by inflammatory stimuli and some cytokines. For example, interleukin (IL)-1 increases the expression of MR in LSECs while IL-10 reduces the activity of the receptor, therefore, MR might be involved in immunity and regulation of glycoprotein homeostasis (Knolle et al., 1998; Arteta et al., 2010). FcγRIIb2, the only Fc gamma-receptor in LSECs, is dedicated to ingesting small soluble immune complexes and cleaning up circulating IgG immune complexes along with KCs (Hebert, 1991; Johansson et al., 1996; Mousavi et al., 2007; Sorensen et al., 2012).

### Immune Regulation of Liver Sinusoidal Endothelial Cells

LSECs are continuously exposed to food and microbial antigens, which come from the gastrointestinal tract and enter hepatic sinusoids through the portal vein (Kubes and Jenne, 2018). As hepatic immune gatekeepers, LSECs maintain a hepatic immune tolerance environment and immune response to cope with other foreign pathogens and thus keep the liver from being damaged by unnecessary immune responses (Kubes and Jenne, 2018; Shetty et al., 2018). Recent studies showed that LSECs participate in both innate and adaptive immune regulation. Pattern recognition receptors including the Toll-like receptor (TLR) family, along with endocytosis receptors, assign LSECs with an ability to recognize and ingest foreign antigens (Chen and Nunez, 2010; Wu et al., 2010; Shetty et al., 2018). Furthermore, LSECs can still respond to signals mediated by these receptors even in the liver immune tolerance environment, though the activation of TLRs in LSECs is relatively limited compared to classical antigen-presenting cells (Wu et al., 2010; Shetty et al., 2018). Besides, endocytosis receptors on LSECs also play an important cell-specific role through interaction with TLRs and regulation of inflammation-related signals (Canton et al., 2013; Shetty et al., 2018). In adaptive immunity, LSECs can cross-present antigen to CD8^+^ T cells by using SRs and induce tolerant naive CD8^+^ T cells through enhanced interaction between programmed cell death one ligand one on LSECs and programmed cell death protein one on CD8^+^ T cells (Limmer et al., 2000; Limmer et al., 2005; Burgdorf et al., 2007; Diehl et al., 2008; Shetty et al., 2018). Certainly, when faced with harmful pathogen stimulation, LSECs can also effectively drive T cells to respond for the rapid elimination of antigens, and this effect is regulated by inflammatory factors (Shetty et al., 2018). LSECs also express major histocompatibility complex class II molecules but are more inclined to promote the differentiation of naive CD4^+^ T cells into regulatory T cells rather than T helper cells (Knolle et al., 1999; Carambia et al., 2014; Shetty et al., 2018).

## The Pro- and Anti-inflammatory Dual Effects of Liver Sinusoidal Endothelial Cells in Nonalcoholic Fatty Liver Disease

**FIGURE 2:**
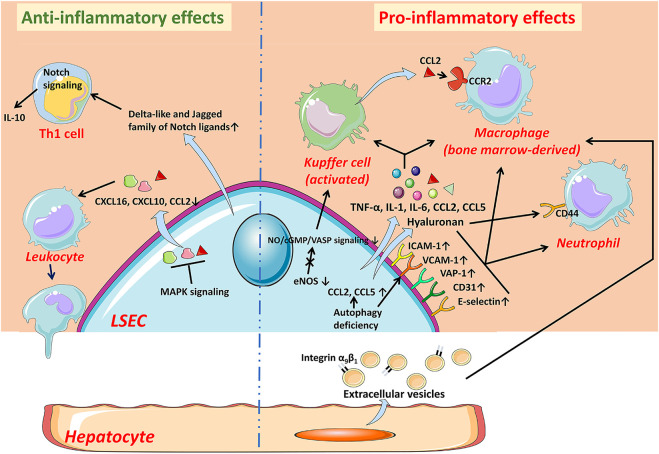
The anti-inflammatory and pro-inflammatory effects of LSECs in NAFLD. In the early stage of NAFLD, LSECs develop an anti-inflammatory phenotype manifested by the decreased expression of chemokines CCL2, CXCL10, and CXCL16 through MAPK signaling-dependent manner. LSECs can also promote the secretion of IL-10 by Th1 cells via releasing Notch ligands to exert anti-inflammatory effects. While LSECs characterized as pro-inflammatory phenotype, increase expression of adhesion factors including VCAM-1, ICAM-1, E-selectin, CD31, and VAP-1, leading to increased recruitment of leukocytes. The deficiency of autophagy also leads to up-regulation of adhesion factors and chemokines. Pro-inflammatory mediators released by LSECs, such as TNF-α, IL-1, and IL-6, also promote the progress of inflammation. The reduction of NO bioavailability contributes to the activation of KCs and the recruitment of bone marrow-derived macrophages. The hyaluronan densely coated on the surface of LSECs facilitates the recruitment of neutrophils by interacting with CD44. In addition, hepatocyte-derived EVs contribute to the recruitment of macrophages into the hepatic sinusoids. *CCL* C-C motif chemokine ligand, *CCR* C-C motif chemokine receptor, *CXCL* C-X-C motif chemokine ligand, *eNOS* endothelial nitric oxide synthase, *ICAM-1* intercellular adhesion molecule-1, *IL* interleukin, *LSEC* liver sinusoidal endothelial cell, *NO* Nitric oxide, *TNF-α* tumor necrosis factor-α, *VAP-1* vascular adhesion protein-1, *VCAM-1* vascular cell adhesion molecule-1.

The development of liver inflammation is a key step causing liver injury, regardless of etiologies (Shetty et al., 2018). In NAFLD, infiltrated leukocytes are recruited from the circulation, mainly bone marrow-derived macrophages (BMMs) and neutrophils, into liver parenchyma, and this leads to the formation of inflammatory foci, and thus accelerates the disease progression from simple steatosis to steatohepatitis (Rinella, 2015). In general, circulating leukocytes are first captured by activated endothelial cells and then migrate through the endothelium to the site of infection or injury (Shetty et al., 2018). The recruitment of leukocytes is an outcome of multi-step adhesion cascades involving various cytokines and receptors on the surface of leukocyte and LSEC (Tanaka et al., 1993; Adams et al., 1996; Wong et al., 1997; Campbell et al., 1998; Nourshargh and Alon, 2014; McEver, 2015; Muller, 2016). In most vascular beds, selectin receptors mediate the capture of circulating leukocytes and cause them to roll on the endothelial surface, which is a general initial step for leukocyte recruitment. However, in hepatic sinusoids, the “rolling” is not an essential step for leukocyte recruitment and thus the selectin may have a minimal function due to the narrow structure and low shear stress (Adams et al., 1996; Wong et al., 1997). Further, stimulated by chemokines, the integrins on the surface of the leukocytes are activated and then mediate a firm adhesion between leukocytes and endothelium. Finally, leukocytes migrate through the endothelium into the liver parenchyma mediated by complex receptor-ligand interactions (Muller, 2016). In most organs, recruitment of leukocytes mainly occurs in post-capillary venules, while in the liver, most leukocyte recruitment occurs in sinusoidal cavities (Shetty et al., 2018). Therefore, LSECs control the key path of inflammation and thus play a pivotal role in the occurrence and progression of liver inflammation (Figure 2).

### Pro-inflammatory Effects of Liver Sinusoidal Endothelial Cells

#### Capillarization of Liver Sinusoidal Endothelial Cells Promotes Inflammation in Early Nonalcoholic Fatty Liver Disease

**FIGURE 3:**
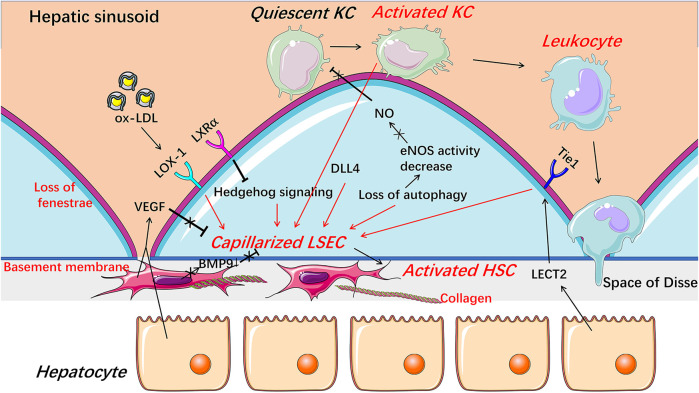
Capillarization and endothelial dysfunction of LSECs under pathological condition. When injured, LSECs undergo capillarization represented by the loss of fenestrae and the formation of basement membrane which are involved in Hedgehog signaling, autophagy, HSCs-derived BMP9, LSECs-derived DLL4, and receptors including LOX-1, LXRα, and Tie1. The reduction of NO bioavailability is an important indicator of endothelial dysfunction and involved in the progress of the capillarization of LSECs. Capillarized and dysfunctional LSECs promote the activation of HSCs and KCs and thus promote liver fibrosis and inflammation. *BMP9* Bone morphogenetic protein 9, *DLL4* Delta-like ligand 4, *HSC* hepatic stellate cell, *KC* Kupffer cell, *LECT2* leukocyte cell-derived chemotaxin 2, *LSEC* liver sinusoidal endothelial cell, *LXRα* liver X receptor α, *LOX-1* lectin-like oxidized low-density lipoprotein receptor-1, *NO* Nitric oxide, *ox-LDL* oxidized low-density lipoproteins, *VEGF* vascular endothelial growth factor.

LSECs undergo morphological changes in specific cases, such as viral infection, chronic liver diseases, and aging (Schaffner and Poper, 1963; Horn et al., 1987; Xu et al., 2003; Warren et al., 2007; Baiocchini et al., 2019; Hunt et al., 2019). Capillarization is the most common and prominent phenotypic change of LSECs, which is mainly manifested by the loss of fenestrae and the formation of a basement membrane on the abluminal surface, and thus it is also called dedifferentiation (Figure 3) (Maslak et al., 2015a). Capillarization of LSECs occurs in lipid-treated LSECs, animal NAFLD models, and patients with NAFLD (Akyol et al., 2005; Peng et al., 2014; Zhang et al., 2014; Miyao et al., 2015; Bravo et al., 2019; Zhang et al., 2019). Treatment with ox-LDL only or plus high glucose induced human LSEC injury and thus caused a reduction in porosity of LSECs (Zhang et al., 2014; Zhang et al., 2019). Shown with a scanning electron microscopy or marker of capillarization indicators such as CD31 and CD34, the capillarization of LSECs was observed at the early stage of NAFLD in animal models induced by diet, including high fat diet (HFD), choline-deficient l-amino acid-defined (CDAA) diet, and high fat glucose-fructose diet (HFGFD) (Table 1) (Peng et al., 2014; Miyao et al., 2015; Bravo et al., 2019). In patients with NAFLD, CD31 was significantly higher expressed in zone 3 (centrilobular area) (Akyol et al., 2005). Certainly, the capillarization of LSECs occurs earlier than the onset of general liver inflammation. Miyao and colleagues found that the reduced porosity of LSECs was earlier than inflammation and activation of KCs and HSCs, which occurred in C57BL/6 mice after 1 week of CDAA diet (Miyao et al., 2015). Straub and colleagues found that CD45/CD68-positive inflammatory cells did not accumulate in the liver until the porosity of LSECs decreased by low-level arsenic, indicating that inflammation may be the consequence of LSECs capillarization (Straub et al., 2007). However, subjected to several variable factors, including the heterogeneity of animal models and LSECs isolated *in vitro*, the difference of detection methods, indicators, and techniques, whether the capillarization of LSECs occurs throughout NAFLD is still controversial.

**TABLE 1:** Summary of capillarization and endothelial dysfunction of LSECs in NAFLD animal models.

Model	Species	Capillarization	Stage	Endothelial dysfunction	Stage	Capillarization Indicator(s)	SEM	AFM	Year	Ref
HFD	C57BL/6 mice	Not mentioned	N/A	Yes	Early (4 weeks)	N/A	No	No	2011	Tateya et al. (2011)
CafD	Wistar kyoto rats	No	Early (1 month)	Yes	Early (1 month)	SE-1, CD31, CD34	No	No	2012	Pasarin et al. (2012)
HFD	SD rat	Yes	Probably early (43rd day)	Not mentioned	N/A	No	Yes	No	2014	Peng et al. (2014)
CDAA	C57BL/6 mice	Yes	Early (1 week)	Not mentioned	N/A	CD31, CD34	Yes	No	2015	Miyao et al. (2015)
HFD	C57BL/6 mice	Yes	Late (22 weeks)	Not mentioned	N/A	No	Yes	No	2015	Miyao et al. (2015)
MCD	C57BL/6 mice	Not mentioned	N/A	Yes	Early (4 weeks)	N/A	No	No	2015	Pourhoseini et al. (2015)
HFGFD	SD OFA rats	Yes	Early (10 weeks)	Yes	Early (10 weeks)	CD31	No	No	2019	Bravo et al. (2019)
HFD	C57BL/6 mice	No	Early (2 weeks) and late (20 weeks)	Not mentioned	N/A	No	Yes	Yes	2019	Kus et al. (2019)

AFM, atomic force microscopy; CafD, cafeteria diet; CDAA, choline-deficient l-amino acid-defined; HFD, high fat diet; HFGFD, high fat glucose-fructose diet; MCD, methionine and choline deficient; N/A, not applicable; SEM, scanning electron microscopy

Intracellular protein expression abnormality or signaling pathway disturbance contributes to capillarization of LSEC (Figure 3). Hedgehog signaling was activated and thus regulated phenotypic changes to form LSEC capillarization, while inhibitors of Hedgehog pathway partially reversed the capillarized LSECs to a healthy differentiated phenotype and completely prevented LSECs from becoming capillarized both *in vitro* and *in vivo* (Xie et al., 2013). Capillarization of LSEC was also inhibited by liver X receptor (LXR) *α* via suppressing Hedgehog signaling (Xing et al., 2016). Although LXR agonists have shown therapeutic potential in anti-inflammatory and anti-fibrotic aspects, whether these beneficial effects are mainly through alleviating the capillarization of LSECs still needs further exploration (Xing et al., 2016). Lectin-like oxidized low-density lipoprotein receptor-1 (LOX-1) mediates capillarization through reactive oxygen species/nuclear factor-κB signaling pathway in LSECs treated with ox-LDL (Zhang et al., 2014). LOX-1 was up-regulated by many pro-inflammatory cytokines, including transforming growth factor-β, IL-1α, IL-1β, IL-6, and tumor necrosis factor-α (TNF-α), indicating that capillarization and inflammatory response may be mutually reinforced in NAFLD (Navarra et al., 2010; Ozturk et al., 2015). Recently, delta-like ligand 4 (DLL4), a ligand of the Notch signaling pathway, was found to mediate the capillarization of LSECs and the vicious circle between fibrosis and pathological sinusoidal remodeling (Chen et al., 2019).

Paracrine signaling is important for maintaining the differentiated phenotype of LSEC (Figure 3). Bone morphogenetic protein 9 (BMP9), as a paracrine factor derived from HSCs, maintains the fenestrated phenotype of LSECs, contributing to liver homeostasis (Desroches-Castan et al., 2019). In patients with NASH, hepatic mRNA expression of BMP9 was decreased (John et al., 2019). Knocking out mouse BMP9 gene induced capillarization of LSECs *in vivo*, while treatment with an addition of BMP9 maintained the fenestration of primary cultured LSECs *in vitro*, and an absence of LSEC fenestrae occurred followed by liver inflammation and fibrosis in BMP9 gene knock-out mice (Miyao et al., 2015; Desroches-Castan et al., 2019). Leukocyte cell-derived chemotaxin 2 (LECT2), a functional ligand of Tie1 expressed by hepatocytes and endothelial cells, promotes the capillarization of LSECs in the liver fibrosis rodent model (Xu et al., 2019). Vascular endothelial growth factor (VEGF), as a paracrine signal produced by hepatocytes and HSCs, maintains the differentiated phenotype of LSECs (DeLeve et al., 2004). When co-cultured with HSCs or hepatocytes *in vitro*, the expression of CD31 on the surface of LSECs was decreased, while anti-VEGF antibody reversed the decrease (DeLeve et al., 2004). Fortuitously, NO synthase inhibitor blocked the stabilizing effect of hepatocytes or HSCs on LSECs phenotype in the co-cultured system, suggesting that NO may be also necessary for maintaining the differentiated phenotype of LSECs and that the decreased NO bioavailability may promote the capillarization of LSECs, which may be associated with endothelial dysfunction of LSECs (DeLeve et al., 2004; Flammer et al., 2012; Hammoutene and Rautou, 2019).

Capillarization of LSECs promotes the development of steatosis in NAFLD. On the one hand, the capillarization of LSECs prevents the release of very-low-density lipoprotein (VLDL) from hepatocytes into the sinusoidal cavity, resulting in the retention of lipids including cholesterol and triglycerides in the liver (Hammoutene and Rautou, 2019). On the other hand, the capillarization of LSECs prevents the entry of chylomicron remnants into hepatocytes, stimulating the *de novo* lipogenesis of liver lipids and eventually inducing steatosis in a compensatory way since the chylomicron remnants are required for the synthesis of VLDL by hepatocytes (Herrnberger et al., 2014; Tanaka and Iwakiri, 2016). However, studies showed that excessive lipid exposure, such as ox-LDL, leads to a decrease in the fenestrae diameter and porosity of LSECs (Zhang et al., 2014). Furthermore, Cogger and colleagues demonstrated that the porosity and fenestrae frequency of LSECs are negatively correlated with dietary fat intake and circulating FFA *in vivo* (Cogger et al., 2016). These data suggested that the early formation of steatosis will, in turn, promotes the capillarization of LSECs, which further facilitates the development of steatosis to inflammation in patients with NAFLD.

#### Dysfunction of Liver Sinusoidal Endothelial Cells Promotes Inflammation in Nonalcoholic Fatty Liver Disease

Endothelial dysfunction of LSECs is a pathological condition mainly characterized by an abnormal imbalance between vascular endothelium-derived relaxing factors and contracting factors, resulting in an inability to expand blood vessels when blood flow increase (Flammer et al., 2012). Much evidence suggest that endothelial dysfunction of LSECs promotes inflammation in NAFLD.

First, endothelial dysfunction of LSECs occurs earlier than liver inflammation in NAFLD (Table 1). Arterial endothelial dysfunction of LSECs was confirmed in patients with NAFLD, and the occurrence of sinusoidal endothelial dysfunction of LSECs was earlier than inflammation in various early stage NAFLD rodent models induced by diet, including cafeteria diet (CafD), HFD, and methionine-choline-deficient (MCD) diet, showing with a marked increased vascular resistance, or increased portal perfusion pressure and reduced endothelium-dependent vasodilatory response (Villanova et al., 2005; Tateya et al., 2011; Francque et al., 2012; Pasarin et al., 2012; Pourhoseini et al., 2015; Gonzalez-Paredes et al., 2016; Bravo et al., 2019). The dysfunction mechanism may be related to a decreased phosphorylation of Akt-dependent endothelial nitric oxide synthase (eNOS) and NO synthase activity, which reduces liver eNOS activity and NO content, respectively, and the consequence was occurred earlier than general liver inflammation in the rodent NAFLD model (Tateya et al., 2011; Pasarin et al., 2012; Gonzalez-Paredes et al., 2016; Bravo et al., 2019).

Second, endothelial dysfunction of LSECs promotes activation of KCs to facilitate the occurrence of inflammation in NAFLD (Lanthier, 2015). KCs are resident macrophages in liver sinusoids that are in close contact with LSECs and can be activated by various factors, including pathogen-associated molecular patterns such as lipopolysaccharide (LPS), damage-associated molecular patterns released by damaged hepatocytes, and lipids such as free fatty acids (FFAs), ceramides, and oxidized lipoproteins (Leroux et al., 2012; Sawada et al., 2014; Zannetti et al., 2016). In NASH patients, an expansion of KCs is an early phenomenon, earlier than the recruitment of other immune cells (Gadd et al., 2014). As a hepatic immune gatekeeper, LSEC maintains KCs quiescence under physiological conditions through NO-dependent pathways (Tateya et al., 2011). However, under pathological conditions, reduced endothelial NO bioavailability promotes the activation of KCs to cause liver inflammation manifested by activation of nuclear factor-κB and upregulation of pro-inflammatory factors TNF-α and IL-6 in mice (Tateya et al., 2011). The activated KCs promote the capillarization of LSECs and lead to subsequent leukocyte recruitment (Arii and Imamura, 2000; Ford et al., 2015; Shetty et al., 2018). Therefore, the dysfunction of LSECs promotes activation of KCs, which in turn contributes to the morphological changes and activation of LSECs, and this interaction leads to exacerbation of liver inflammatory response in the early stage of NAFLD. Certainly, ameliorating endothelial dysfunction of LSECs can improve liver inflammation in animal models by manipulating the NO signal pathway using sildenafil or simvastatin (Tateya et al., 2011; Wang et al., 2013; Ahsan et al., 2020).

Third, under pathological conditions, autophagy of LSECs is often abnormal. Autophagy, a major intracellular recycling system, maintains cellular homeostasis under basal conditions and acts as a survival mechanism under stress conditions (Hammoutene et al., 2020). Defect autophagy with smaller autophagic vacuoles in LSECs occurs in patients with NASH. The endothelial cell-specific loss of autophagy leads to a significant decrease in porosity and the number of fenestrae of LSECs in mice after mild acute liver injury and an increase in the expression of pro-inflammatory chemokines C-C motif chemokine ligand (CCL) 2, CCL5, and vascular cell adhesion molecule (VCAM-1), and thus promotes inflammation in mice fed with HFD (Ruart et al., 2019; Hammoutene et al., 2020). Interestingly, in different cell types, autophagy may play differential roles in chronic liver diseases (Choi et al., 2013; Allaire et al., 2019). For example, in hepatocytes, autophagy helps to protect cells from fat accumulation and prevents liver damage by removing altered mitochondria and reducing intracellular stresses. In macrophages, it has anti-inflammatory effects and thus prevents liver inflammation and fibrosis. While in activated HSCs and LSECs, autophagy promotes liver disease progression (Berg et al., 2006; Hernandez-Gea et al., 2012; Lodder et al., 2015; Madrigal-Matute and Cuervo, 2016; Gual et al., 2017; Gracia-Sancho and Guixe-Muntet, 2018; Hammoutene et al., 2020). Therefore, the specific mechanism of autophagy abnormality in LSECs leading to capillarization and inflammation still needs more exploration.

The dysfunction of LSECs promotes steatosis by increasing intrahepatic vascular resistance, which is related to the formation of steatosis (Tateya et al., 2011; Francque et al., 2012; Gonzalez-Paredes et al., 2016; Baffy, 2018). NO plays an important role in regulating liver lipid content and fatty acid synthesis (Roediger et al., 2004; Schild et al., 2008; Tateya et al., 2011; Doulias et al., 2013). In NO-deficient eNOS knockout mice, large amounts of lipid droplets and elevated liver triglyceride levels were observed, while treatments with increasing the availability of NO improved not only liver inflammation but also liver steatosis (Schild et al., 2008; Tateya et al., 2011). However, steatosis, in turn, promotes the dysfunction of LSECs. The mechanism may be that excess lipids of steatosis decreased eNOS expression and thus reduced the bioavailability of NO (Pasarin et al., 2011; Pasarin et al., 2012; Pasarin et al., 2017). Besides, insulin resistance caused by steatosis can damage the vasodilation function of LSECs through the decrease of eNOS and the increase of inducible nitric oxide synthase (iNOS) (Chauhan et al., 2003; Pasarin et al., 2017).

#### Liver Sinusoidal Endothelial Cells Promote Inflammation by the Recruitment of BMMs and Neutrophils in Nonalcoholic Steatohepatitis

Recruited BMMs are an important component of chronic liver inflammation in NASH, where LSECs play a crucial role in the recruitment of BMMs (Koyama and Brenner, 2017). The dysfunction of LSECs facilitates the recruitment of macrophages in NASH through promoting KCs activation to release CCL2, which is the ligand of C-C motif chemokine receptor (CCR) two mainly expressed on monocytes and macrophages and thus is critical for the recruitment of macrophages (Tateya et al., 2011; Miura et al., 2012). Furthermore, LSECs also promote the recruitment of leukocytes, including but not limited to BMMs, directly by increasing the expression of adhesion molecules including intercellular adhesion molecule-1 (ICAM-1), VCAM-1, vascular adhesion protein-1 (VAP-1), E-selectin, and CD31, which are essential for the interaction between LSECs and leukocytes to recruit leukocytes (Edwards et al., 2005; Weston et al., 2015; Miyachi et al., 2017; Hammoutene and Rautou, 2019; Kus et al., 2019). In addition to this pro-inflammatory phenotype, LSECs produce pro-inflammatory mediators, including IL-1, IL-6, TNF-α, and CCL2, to promote activation of inflammatory cells and the recruitment, adhesion, and migration of BMMs and neutrophils in NASH (Miyachi et al., 2017; Roh and Seki, 2018; Hammoutene and Rautou, 2019).

Recent research shows that the recruitment of BMMs by LSECs is partly influenced by extracellular vesicle (EV) derived from hepatocytes. EV is a general term for membrane vesicles released by cells, it plays an important role in liver physiology and pathology, which has been reviewed well elsewhere (Hirsova et al., 2016b; Eguchi and Feldstein, 2018). In NASH, EVs released by hepatocytes under pressure stimulation contribute to the occurrence of liver inflammation by inducing pro-inflammatory cytokines expression and activating macrophage chemotaxis and thus promoting macrophages recruitment (Hirsova et al., 2016a; Garcia-Martinez et al., 2016; Ibrahim et al., 2016; Kakazu et al., 2016). Recently, Guo and colleagues found that integrin *α*_9_β_1_-enriched EVs released by lysophosphatidylcholine-treated hepatocytes interact with monocytes in a topography and assist monocytes to adhere to LSECs *in vivo* and *in vitro* (Guo et al., 2019). However, the influence of other cell-derived EVs on LSECs and the effect of LSECs-derived EVs on macrophage recruitment in NASH require more researches.

Besides BMMs, neutrophil infiltration is also commonly observed in patients with NAFLD, and its severity is associated with the development of the disease (Nati et al., 2016; Cai et al., 2019). In the inflammatory state, neutrophils upregulate the expression of adhesion molecules and activate endothelial cells and KCs, which induce the further recruitment of other leukocytes including BMMs, where neutrophils are excessively activated and release proteases such as myeloperoxidase causing liver damage, and thus aggravate the ongoing inflammatory state (Arrese et al., 2016; Nati et al., 2016; Cai et al., 2019). In the inflamed hepatic sinusoids, highly coated hyaluronan on the luminal surface of LSECs interacts with CD44 on the surface of neutrophils and thus mediates the recruitment of neutrophils (McDonald et al., 2008). Unlike other vascular endothelial cells, LSEC anchoring hyaluronan does not depend on the endothelial CD44. Instead, LSECs capture the circulating hyaluronan through a variety of SRs such as stabilin-2 on the cell surface and then present it to the passing neutrophils before promoting the endocytosis of hyaluronan ultimately (McDonald and Kubes, 2015). Therefore, it is necessary to study the role of SRs on LSECs in LSECs-mediated leukocyte recruitment.

### Anti-inflammatory Effects of Liver Sinusoidal Endothelial Cells in Nonalcoholic Fatty Liver Disease

#### Liver Sinusoidal Endothelial Cells Prevent Formation of Inflammation by Inhibiting Leukocyte Recruitment in the Early Stage of Nonalcoholic fatty liver disease

In the early stage of NAFLD models, evidence suggest that LSECs suppress leukocyte recruitment into hepatic sinusoids (McMahan et al., 2016). An anti-inflammatory phenotype of LSECs, characterized by decreased expressions of CCL2, C-X-C motif chemokine ligand (CXCL) 10, and CXCL16, was produced in both murine and human LSECs after a short time exposure to FFA, and thus reduced the recruitment of pro-inflammatory monocytes. Primary LSECs isolated from obese mice also showed the consequence (McMahan et al., 2016). Further study demonstrated the anti-inflammatory ability produced by LSECs under FFA induction depends on the MAPK signaling pathway, which is important for the survival of LSECs in the case of lipid induction, and it also may be involved with signal transducer and activator of transcription 3 (STAT3) for its expression in LSECs alleviated mice liver inflammation induced by alcohol (Miller et al., 2010; Hang et al., 2012; McMahan et al., 2016).

#### Liver Sinusoidal Endothelial Cells Regulate Lymphocytes to Exert Anti-inflammatory Effects

LSECs regulate a behavior of lymphocytes under both physiological and pathological conditions (Poisson et al., 2017). Under physiological conditions, LSECs maintain the intrahepatic tolerance environment through inducing tolerant CD8^+^ T cell and immunosuppressed regulatory T cells (Limmer et al., 2000; Carambia et al., 2014). While under inflammatory conditions, LSECs express high levels of Delta-like and Jagged family of Notch ligands and induce the expression of Notch target genes in Th1 cells, by which increases the expression of inflammatory cytokine IL-10 in Th1 cells to exert an anti-inflammatory effect (Neumann et al., 2015).

## Therapeutic Perspectives

Currently, no specific drug has been approved for clinical use to treat patients with NASH. There are many promising candidates in the drug development pipeline, and some have shown very useful for improving NASH by controlling inflammation in clinical trials (Table 2), such as dual CCR2/5 inhibitor, apoptosis signal-regulating kinase 1 (ASK1) inhibitor, and caspase inhibitor (Romero et al., 2020). Unfortunately, the latest clinic outcomes of selonsertib (an ASK1 inhibitor) and emricasan (a pan-caspase inhibitor) are not satisfied (Loomba et al., 2018; Harrison et al., 2020; Ratziu et al., 2020; Romero et al., 2020). Thus, it is still urgent and important to find new anti-inflammatory targets in NASH. Most encouragingly, the essential role of LSECs in liver inflammation provides new insights into the development of treatment strategies for NAFLD/NASH.

**TABLE 2:** Inflammation-targeted pharmacologic agents that have completed or are undergoing clinical trials for NASH.

Mechanism of action	Agent name	Company(s)	Trial phase	Primary endpoint(s)	Primary completion	Clinical trial ID
CCR2/5 inhibitor	Cenicriviroc	Allergan	3	1. Improvement in fibrosis without worsening steatohepatitis; 2. Long-term clinical outcomes	Oct 2021	NCT03028740
ASK1 inhibitor	Selonsertib	Gilead	3	1. Improvement in fibrosis without worsening of NASH; 2. Event-free survival	Jun 2019	NCT03053050
			3	1. Improvement in fibrosis without worsening of NASH; 2. Event-free survival	May 2019	NCT03053063
Pan-caspase inhibitor	Emricasan	Novartis/Conatus pharmaceuticals	2	Improvement in fibrosis without worsening of steatohepatitis	Jan 2019	NCT02686762
			2	Improvement in event-free survival based on a composite clinical endpoint	Aug 2019	NCT03205345
TLR-4 antagonist	JKB-121	TaiwanJ	2	Change from baseline in hepatic fat	Sept 2017	NCT02442687
	JKB-122	TaiwanJ	2	1. Reduction in NAS without worsening of fibrosis; 2. Improvement in fibrosis without worsening of NAS	Jun 2023	NCT04255069
Mineralocorticoid receptor antagonist	MT-3995	Mitsubishi tanabe pharma	2	Percent change from baseline in ALT	Mar 2018	NCT02923154
VAP-1 inhibitor	BI-1467335	Boehringer ingelheim	2	Target enzyme activity relative to baseline in percent	Jun 2019	NCT03166735
	TERN-201	Terns pharmaceuticals	1	Safety and tolerability		

ALT, alanine aminotransferase; ASK1, apoptosis signal-regulating kinase 1; CCR2/5, C-C motif chemokine receptor 2/5; NAS, non-alcoholic fatty liver disease activity score; NASH, non-alcoholic steatohepatitis; TLR-4, toll-like receptor-4; VAP-1, vascular adhesion protein-1.

### Targeting Adhesion-Related Molecules to Alleviate Inflammation in Nonalcoholic Steatohepatitis

Adhesion molecules are abnormally expressed on LSECs in response to liver injury and regulate inflammation via corresponding ligands. Therefore, adhesion molecules and their ligands related to LSECs are provided with multiple potential targets to control inflammation in NASH. The pro-inflammatory phenotype of LSECs showed increased expression of adhesion molecules including VAP-1, VCAM-1, CD31, ICAM-1, and E-selectin (Hammoutene and Rautou, 2019; Kus et al., 2019). Blocking these molecules or their ligands is efficacious to control inflammation in various NASH models (Edwards et al., 2005; Weston et al., 2015; Miyachi et al., 2017; Hammoutene and Rautou, 2019). In particular, VAP-1 inhibitors have entered clinical trials. However, unfortunately, a VAP-1 inhibitor BI 1467335 was recently discontinued for NASH indications due to its interaction with other drugs, although the results of the latest clinical studies (phase IIa, NCT03166735) did not indicate a direct failure of BI 1467335 in terms of efficacy and tolerance (Boehringer ingelheim, 2019). Another potent VAP-1 inhibitor, TERN-201, is still undergoing clinical trials in China for the treatment of NASH (Terns Pharmaceuticals., 2019). Blocking the interaction between the adhesion molecule CD44 and its ligand hyaluronan using an anti-CD44 antibody also exhibits a potential inflammation-controlling effect both in LPS-induced or diet-induced mouse models, but further research is still needed in NASH (McDonald et al., 2008; Kodama et al., 2015; McDonald and Kubes, 2015).

Chemokines are chemo-attractants for leukocyte trafficking, growth, and activation in injured and inflammatory tissues (Roh and Seki, 2018). They are also abnormally expressed and secreted by LSECs under pathological conditions. The anti-inflammatory phenotype of LSECs showed decreased expression of chemokines including CXCL10, CXCL16, and CCL2 (McMahan et al., 2016). Hopefully, the dual CCR2/5 inhibitor cenicriviroc was well tolerated in NASH patients and is currently undergoing a phase III clinical trial (NCT03028740) (Ratziu et al., 2020).

### Targeting Nitric Oxide Signaling to Improve Nonalcoholic fatty liver disease

LSECs are the major producers of NO in the liver (Shah et al., 1997). The balance of NO is critical in maintaining the morphology and endothelial function of LSECs to keep the quiescence of HSCs and KCs, it also fundamentally participates in the regulation of liver lipid and glucose homeostasis (Maslak et al., 2015a). Therefore, targeting NO-related signaling is an attractive therapeutic strategy to improve liver inflammation and alleviate liver damage. Some efforts to improve the bioavailability of NO in NAFLD have been made. V-PYRRO/NO, a stable hepato-selective NO-releasing prodrug, improved liver steatosis and postprandial glucose tolerance in NAFLD mice fed HFD (Maslak et al., 2015b). Praliciguat, a soluble guanylate cyclase stimulator, effectively reduced inflammation, fibrosis, and steatosis by enhancing NO signaling in preclinical NASH models (Hall et al., 2019). Besides, improvement of the NO/cGMP signaling pathway by using phosphodiesterase-5 inhibitor sildenafil or simvastatin prevented liver inflammation in NAFLD rodents fed HFD (Tateya et al., 2011; Wang et al., 2013; Ahsan et al., 2020).

### Targeting Angiogenesis to Improve Nonalcoholic fatty liver disease

Hepatic angiogenesis, including the capillarization of LSECs, is an important event in the progression of NAFLD, especially in the formation of hepatic fibrosis (Coulon et al., 2011; Coulon et al., 2012; Coulon et al., 2013; Iwakiri et al., 2014). New blood vessels are produced in the liver of NASH patients, but not in individuals with simple steatosis or healthy liver (Kitade et al., 2008; Kitade et al., 2009; Lefere et al., 2019). In the serum of patients with NASH, the level of VEGF, a major pro-angiogenesis regulator, was increased significantly (Yoshiji et al., 2006; Coulon et al., 2011; Tamaki et al., 2013; Lefere et al., 2019). Evidence of abnormal angiogenesis was also found in animal models of NASH (Coulon et al., 2013).

A variety of anti-angiogenic therapies have shown anti-inflammatory effects in NASH animal models. Coulon and colleagues used specific antibodies to block vascular endothelial growth factor receptor 2 (VEGFR2) and found that liver inflammation and liver vasculature were significantly improved in the MCD-induced mouse NASH model, both in a preventive and therapeutic setting (Coulon et al., 2013). Studies have shown that serum level of angiopoietin-2, a key factor involved in regulating angiogenesis, is elevated in patients with NASH (Lefere et al., 2019). Inhibiting the interaction between angiopoietin-2 and its receptor Tie2 by using peptibody L1-10 effectively improved liver inflammation and damage in the NASH model induced by MCD (Lefere et al., 2019). It is worth noting that the therapeutic effect of L1-10, at least in part, is mediated by LSECs as evidenced by the downregulation of VCAM-1, ICAM-1, and CCL2 expression in liver endothelial cells isolated from the liver of NASH mouse (Lefere et al., 2019). Besides, LECT2, a functional ligand of endothelial cell-specific receptor Tie1, was recently found to promote liver fibrosis by inhibiting portal angiogenesis and promoting capillarization of liver sinusoids in various liver fibrosis models, providing a novel possible target for LSECs-mediated liver fibrosis (Xu et al., 2019). Therefore, studies on angiogenesis in the liver including capillarization of LSECs may provide new targets for NASH treatment.

### Targeting Pro-inflammatory EVs Specifically to Improve Inflammation

Hepatocyte-derived EVs exert a regulatory ability in the recruitment of leukocytes to hepatic sinusoids. Therefore, blocking the production pathway of pro-inflammatory EVs or targeting pro-inflammatory cargos carried by EVs may improve liver inflammation in NASH, though the research in this area has only just begun (Hirsova et al., 2016a; Garcia-Martinez et al., 2016; Ibrahim et al., 2016; Kakazu et al., 2016; Guo et al., 2019). Using fasudil, an inhibitor of Rho-associated coiled coil-containing protein kinase one that is required for membrane blebbing, to reduce the serum levels of hepatocytes-derived EVs, or using anti-integrin *α*_9_β_1_ antibody alleviated liver damage, inflammation, and fibrosis in diet-induced NASH model (Hirsova et al., 2016a; Guo et al., 2019). However, EVs are widely involved in human physiology, and their cargos play different roles in different environments. Therefore, how to achieve cell- or tissue-specific targeting of EVs will be the focus of future research.

### Promoting Anti-inflammatory Behavior of Liver Sinusoidal Endothelial Cells to Improve Inflammation

As the recruiter of BMMs and neutrophils, LSECs also have compensatory anti-inflammatory behaviors. On the one hand, LSECs can resist the recruitment of leukocytes through the production of anti-inflammatory phenotype, which is mainly manifested as a decrease in the expression of chemokines (McMahan et al., 2016). Therefore, increasing the ratio of the anti-inflammatory phenotype to the pro-inflammatory phenotype of LSECs may be an effective strategy to improve inflammation. However, the mechanism still needs to be further demonstrated that how LSECs switch their phenotype into anti-inflammatory phenotype during the occurrence and development of NAFLD inflammation. On the other hand, LSECs can drive lymphocytes to down-regulate inflammation. Under inflammatory conditions, LSECs release Notch ligands and thereby facilitate Th1 cells to secrete IL-10, an anti-inflammatory cytokine (Neumann et al., 2015). The clue indicates that effectively controlling the behavior of LSECs to indirectly regulate inflammation may be a potential research direction.

## Conclusion

**FIGURE 4:**
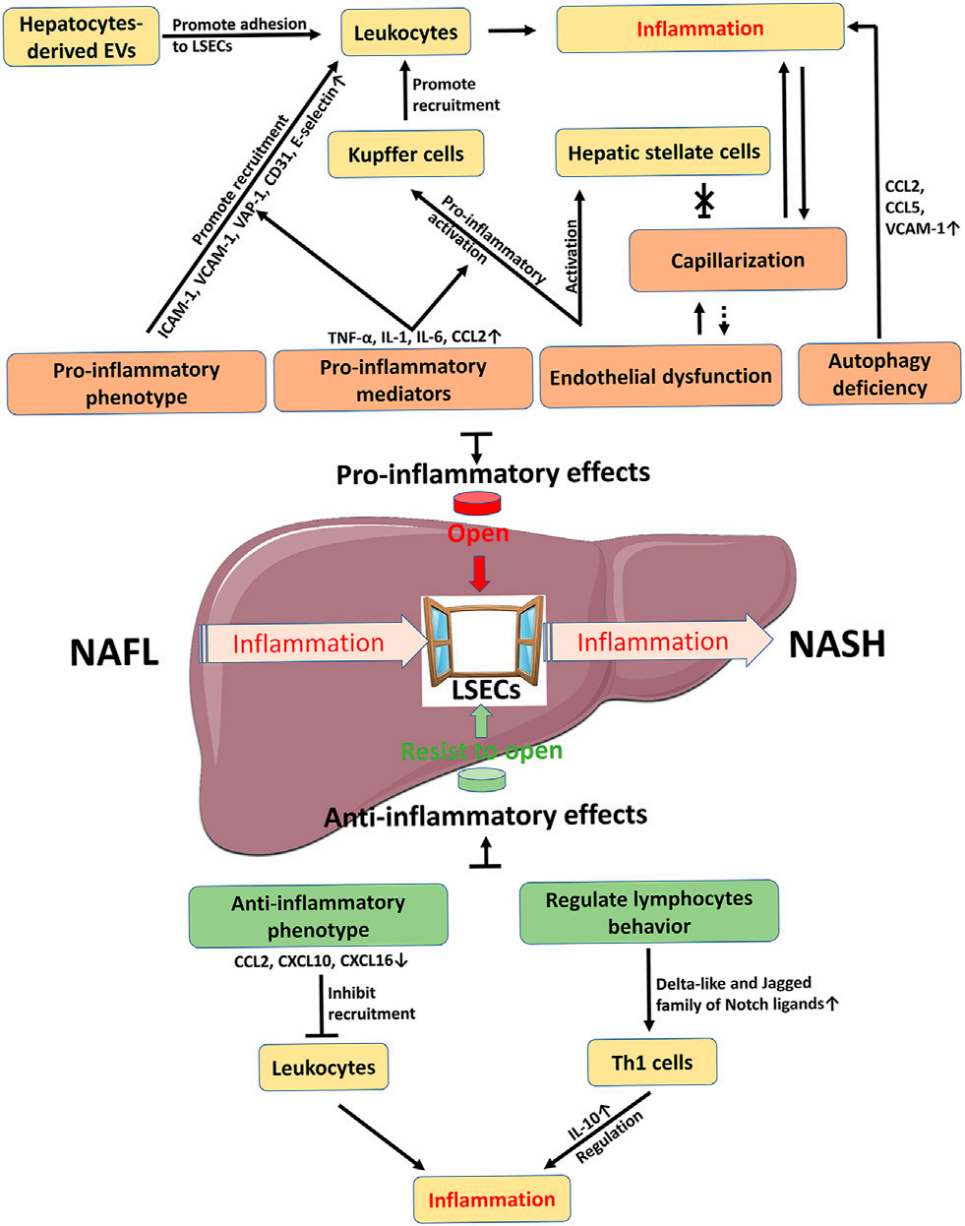
Schematic summary on the role of LSECs in NAFLD inflammation. Inflammation is a key factor in the development of NAFL to NASH. LSEC as a window controls the occurrence and progression of inflammation. The pro-inflammatory effect of LSECs promotes the opening of the window, while the anti-inflammatory effect of LSECs acts as an opponent to prevent the passage of inflammation. Therefore, how to control the window provides new strategies for the R&D of novelty drugs against NAFLD/NASH. The dotted line is a possible relationship. *CCL* C-C motif chemokine ligand, *CXCL* C-X-C motif chemokine ligand, *EVs* extracellular vesicles, *ICAM-1* intercellular adhesion molecule-1, *IL* interleukin, *LSECs* liver sinusoidal endothelial cells, *NAFL,* nonalcoholic fatty liver, *NASH* nonalcoholic steatohepatitis, *TNF-α* tumor necrosis factor-α, *VAP-1* vascular adhesion protein-1, *VCAM-1* vascular cell adhesion molecule-1.

Under physiological conditions, LSECs have multiple functions due to their unique structure and anatomical position, including substance exchange and clearance, blood flow regulation, and immune regulation, and therefore LSECs contribute to the maintenance of liver homeostasis. In NAFLD, LSECs have dual roles in inflammation (Figures 2, 4). On one hand, LSECs block the occurrence of inflammation by generating anti-inflammatory phenotype and regulating lymphocyte behavior. On the other hand, LSECs show pro-inflammatory effects including promoting the activation of KCs and the recruitment of leukocytes. The detailed mechanisms are involved with multiple alterations of LSECs, including morphology and endothelial function, paracrine and autocrine signals, hepatocyte-derived EVs, and autophagy abnormalities. Accordingly, changing these abnormalities of LSECs with new drug candidates is an attractive therapeutic strategy to control inflammation in NAFLD/NASH by regulating LSECs via several variable factors, including adhesion molecules and chemokines expressed by LSECs, capillarization and endothelial dysfunction of LSECs, and other regulatory factors such as EVs. However, the current challenge in this research area is still to clarify the mechanism of LSEC alteration in NAFLD and to further validate the clinical efficacy with those drug candidates specifically targeted to LSEC-related molecules.
